# Modeling clinical trajectory status of critically ill COVID-19 patients over time: A method for analyzing discrete longitudinal and ordinal outcomes

**DOI:** 10.1017/cts.2022.393

**Published:** 2022-04-25

**Authors:** Michael J. Ward, David J. Douin, Wu Gong, Adit A. Ginde, Catherine L. Hough, Matthew C. Exline, Mark W. Tenforde, William B. Stubblefield, Jay S. Steingrub, Matthew E. Prekker, Akram Khan, D. Clark Files, Kevin W. Gibbs, Todd W. Rice, Jonathan D. Casey, Daniel J. Henning, Jennifer G. Wilson, Samuel M. Brown, Manish M. Patel, Wesley H. Self, Christopher J. Lindsell, for the Influenza and Other Viruses in the Acutely Ill (IVY) Network

**Affiliations:** 1 Vanderbilt University Medical Center, Nashville, Tennessee, USA; 2 University of Colorado School of Medicine, Aurora, Colorado, USA; 3 Oregon Health & Science University Hospital, Portland, Oregon, USA; 4 Ohio State University Wexner Medical Center, Columbus, Ohio, USA; 5 CDC COVID-19 Response Team, Atlanta, Georgia, USA; 6 Baystate Medical Center, Springfield, Massachusetts, USA; 7 Hennepin County Medical Center, Minneapolis, Minnesota, USA; 8 Wake Forest University Baptist Medical Center, Winston-Salem, North Carolina, USA; 9 University of Washington School of Medicine, Seattle, Washington, USA; 10 Stanford University School of Medicine, Palo Alto, California, USA; 11 Intermountain Medical Center and University of Utah, Salt Lake City, Utah, USA

**Keywords:** COVID, critical illness, proportional odds, longitudinal assessment, Clinical Progression Scale

## Abstract

Early in the COVID-19 pandemic, the World Health Organization stressed the importance of daily clinical assessments of infected patients, yet current approaches frequently consider cross-sectional timepoints, cumulative summary measures, or time-to-event analyses. Statistical methods are available that make use of the rich information content of longitudinal assessments. We demonstrate the use of a multistate transition model to assess the dynamic nature of COVID-19-associated critical illness using daily evaluations of COVID-19 patients from 9 academic hospitals. We describe the accessibility and utility of methods that consider the clinical trajectory of critically ill COVID-19 patients.

## Introduction

The COVID-19 pandemic spurred rapid investigation to understand and predict the severity of disease among infected patients. Given the major gap in understanding of the clinical course and subsequent disease progression, the World Health Organization (WHO) emphasized daily clinical assessments of patients with COVID-19 [1]. Understanding the trajectory of rapidly progressing diseases might inform the timing of intervention, scientifically relevant endpoints for clinical trials, and help with resource capacity management such as hospital and intensive care unit (ICU) bed capacity [2].

Despite the potential advantages of full information on clinical trajectories, most trials have compared treatment groups at cross-sectional timepoints, e.g., 14 or 30 days after randomization, have used ordered summary measures like ventilator-free days, or time-to-event analyses (e.g., time to recovery). Such approaches do not consider the rich information content arising from the dynamic nature of disease. Statistical methods that model dynamic outcomes have been available for some time [3], and the current pandemic offers the opportunity to demonstrate their real-world application.

**Fig. 1.**
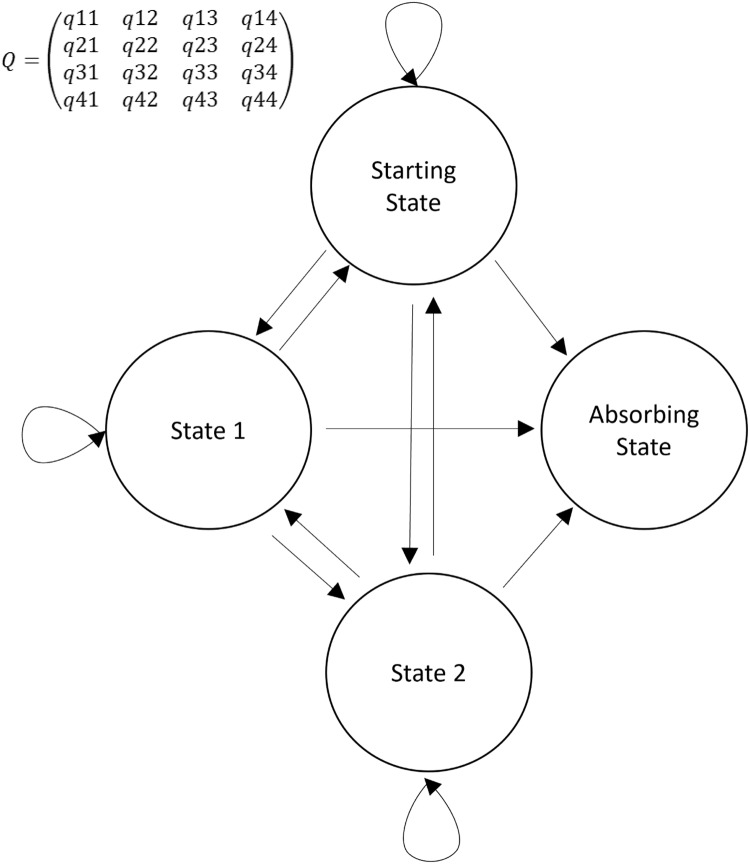
Simplified representation of a Markov transition model. Four states are represented: 1) starting state; 2) two transitional states; and 3) absorbing state. Arrows represent the direction of a transition. Circular arrows represent a transition to the same state. In the transition matrix (Q), the intensity reflects the frequency with which the specific transition is observed. For example, q12 represents the transition intensity (hazard) from state 1 to state 2 and covariates in the model change the magnitudes (hazard ratios) of these intensities (hazards).

Here, we describe the use of a state transition model to evaluate the daily course of critically ill COVID-19 patients. Widely used as a modeling approach in decision analyses and economic evaluations, state transition models conceptualize a series of potential health states and the transitions that occur between them over time [4]. Such a transition model can be simplified in the four states (Fig. 1): one starting state, two transitional states, and one absorbing state. An absorbing state is one from which patients cannot subsequently transition. A sample trajectory could be represented as a critically ill patient requiring mechanical ventilation entering the model (Starting state). On Day 1, they recover and are placed on noninvasive ventilation (State 1). On Day 2, they remain in the same state, and on Day 3, they recover and no longer require noninvasive ventilation. On Day 4, they no longer require critical care and are discharged from the ICU (absorbing state). The multistate model assumes that there is an instantaneous risk of moving between states, as represented, respectively, in the transition intensities matrix Q (Fig. 1).

Using this approach, we demonstrate how baseline demographic and clinical characteristics contribute to the trajectory of disease. We propose that such modeling approaches increase the efficiency with which information is derived from clinical research by better capturing dynamic changes in patient condition.

## Methods

We collected data in real time through surveillance of patients with COVID-19 admitted to nine ICUs across the United States between March and July 2020 that were part of the CDC-funded Influenza and Other Viruses in the Acutely Ill (IVY) network [2,5,6]. Patients were included if they tested positive for SARS-CoV-2 and were admitted to an ICU at any time during their index hospitalization. Through chart abstraction using standardized case report forms, baseline demographics and comorbidities were obtained, and a clinical status scale was assessed daily from ICU admission until either discharge from the ICU, death, or 28 days in the ICU. The five-level scale included 1) discharged alive from the ICU, 2) no supplemental oxygen but still in the ICU, or supplemental oxygen therapy by standard mask or nasal cannula but still in the ICU, 3) high-flow nasal cannula or noninvasive ventilation (continuous positive airway pressure or bilevel positive airway pressure ), 4) invasive mechanical ventilation or extracorporeal membrane oxygenation (ECMO) and 5) death. Patients were classified each calendar day based on the highest level reached for that day. A state transition was defined as movement on the five-level clinical scale from one day to the next consecutive day during the ICU admission. We excluded patients for whom ICU data were not available or who had an ICU length of stay of one day or less as no state transition could occur.

### Statistical model

We constructed a multistate Markov transition model to describe the individual patient’s process of transition through the 5 ordinal states over a single ICU stay. Readmissions were treated as new ICU stays [7]. Discharge from the ICU and death were considered absorbing states. For patients not in absorbing states there are three types of transitions: 1) *disease progression,* in which their daily status worsens (e.g., conventional supplemental oxygen to ECMO), 2) *disease recovery*, when a patient’s daily status improves (e.g., mechanical ventilation to supplemental oxygen), or 3) *unchanged*, when a patient’s clinical status is unchanged from the prior day. Throughout an ICU stay, multiple daily transitions were possible.

From chart review, we included the following covariates: sex, age group (18–49, 50–64, 65+ years), race-ethnicity group (non-Hispanic White, non-Hispanic Black, Hispanic, and Other), and presence of any of 10 comorbidities (asthma, chronic obstructive pulmonary disease, stroke, coronary artery disease, diabetes mellitus, obesity, hypertension, chronic kidney disease, heart failure, or immunosuppression). Each comorbidity was treated as a binary variable for the historical presence or absence of that condition prior to admission. To reduce the number of covariates in the multistate transition model, we applied a restrained proportional hazard model. While there was a unique base transition intensity for every transition between two states, we assume all covariates have the same effects (hazard ratios) on all progressive and recovery transitions. In other words, our model estimated one average hazard ratio for each covariate effect on either all progressive transitions or all recovery transitions. This parsimonious model would be most indicative of clinical decision-making about potential transition status (progression vs. recovery) for the next day. All analyses were conducted in R version 4.0.3 [8], and the multistate model was applied using the msm package [9]. This activity was reviewed by CDC and was conducted consistent with applicable federal law and CDC policy (see e.g., 45 C.F.R. part 46.102(l)(2), 21 C.F.R. part 56; 42 U.S.C. Sect. 241(d); 5 U.S.C. Sect. 552a; 44 U.S.C. Sect. 3501 et seq.).

## Results

We included 514 patients representing 516 ICU admissions from March to July 2020. The two repeat ICU admissions were considered as independent events in the demonstration analysis; the lack of systematic capture of states after ICU discharge precluded inclusion of return to the ICU as a meaningful transition. The cohort is described in Table 1. There were between 1 and 152 cases at each of the nine sites. Participants had a median age of 61.7 (interquartile range [IQR] 50.2–71.3) years, 210 (41%) were aged 65 years or older, 166 (32%) were women, 169 (33%) were non-Hispanic White, 133 (26%) were non-Hispanic Black, and 158 (31%) were Hispanic of any race. Among the 516 admissions, 338 (66%) received mechanical ventilation and/or ECMO at some point during their ICU stay. Overall, 134 (26%) died during their ICU stay. The median ICU length of stay was 8 (IQR 3–17) days. We observed a total of 5,607 transitions. Among the three potential statuses for each transition (disease progression, disease recovery, and unchanged), there were 292 (5.2%) disease progressions from 222 unique patients, 469 (8.4%) recoveries from 351 unique patients, and the remainder were unchanged.

**Table 1.** Demographics of the COVID-19 intensive care unit (ICU) stay cohort in nine participating hospitals, March–July 2020

Variable	Age Group			Overall
	18–49	50–64	65+	
	N = 128	N = 178	N = 210	N = 516
**Sex**				
Female	39 (30.5)	48 (27.0)	79 (37.6)	166 (32.2)
Male	89 (69.5)	130 (73.0)	131 (62.4)	350 (67.8)
**Age at ICU Admission, Median (IQR) years**				
	41.8 (33.5, 46.3)	58.1 (55.1, 62.0)	73.5 (68.2, 79.3)	61.7 (50.2, 71.3)
**Race and Ethnicity, N (%)**				
Non-Hispanic White	18 (14.1)	57 (32.0)	94 (44.8)	169 (32.8)
Non-Hispanic Black	20 (15.6)	54 (30.3)	59 (28.1)	133 (25.8)
Hispanic	64 (50.0)	57 (32.0)	37 (17.6)	158 (30.6)
Others	26 (20.3)	10 (5.6)	20 (9.5)	56 (10.9)
Most Severe Daily State*, N (%)				
No Oxygen	8 (6.2)	4 (2.2)	7 (3.3)	19 (3.7)
Supplemental Oxygen	19 (14.8)	17 (9.6)	26 (12.4)	62 (12.0)
Noninvasive Ventilation	19 (14.8)	32 (18.0)	20 (9.5)	71 (13.8)
Mechanical Ventilation	57 (44.5)	75 (42.1)	69 (32.9)	201 (39.0)
ECMO	14 (10.9)	11 (6.2)	4 (1.9)	29 (5.6)
Death	11 (8.6)	39 (21.9)	84 (40.0)	134 (26.0)
**Admission Month, N (%)**				
March/April	14 (10.9)	27 (15.2)	25 (11.9)	66 (12.8)
May	79 (61.7)	110 (61.8)	121 (57.6)	310 (60.1)
June/July	35 (27.3)	41 (23.0)	64 (30.5)	140 (27.1)
**Comorbidity, N (%)**				
Asthma	14 (10.9)	19 (10.7)	16 (7.6)	49 (9.5)
Coronary Artery Disease	1 (0.8)	18 (10.1)	42 (20.0)	61 (11.8)
Immunosuppression	5 (3.9)	14 (7.9)	16 (7.6)	35 (6.8)
Diabetes	36 (28.1)	85 (47.8)	93 (44.3)	214 (41.5)
Hypertension	38 (29.7)	111 (62.4)	159 (75.7)	308 (59.7)
Stroke	3 (2.3)	15 (8.4)	36 (17.1)	54 (10.5)
Chronic Kidney Disease	11 (8.6)	22 (12.4)	56 (26.7)	89 (17.2)
COPD	1 (0.8)	20 (11.2)	49 (23.3)	70 (13.6)
Heart Failure	2 (1.6)	20 (11.2)	39 (18.6)	61 (11.8)
Obesity	53 (41.4)	83 (46.6)	59 (28.1)	195 (37.8)
**ICU Length of Stay, Median (IQR) days**	8.0 (2.8, 17.0)	11.0 (4.0, 19.0)	6.0 (3.0, 14.0)	8.0 (3.0, 17.0)
**Transition** ^**†**^ **, N (%)**				
Experienced Progression	39 (30.5)	77 (43.3)	106 (50.5)	222 (43.0)
Experienced Recovery	103 (80.5)	118 (66.3)	130 (61.9)	351 (68.0)

* Categories presented are mutually exclusive and represent the most severe clinical state on 1 or more days during ICU admission, e.g., if patient died in the ICU but received invasive mechanical ventilation before death they are classified as “Death.”

^†^ Transition states add up to > 100% as a patient could experience both recovery and progression transitions during ICU admission.

**Fig. 2.**
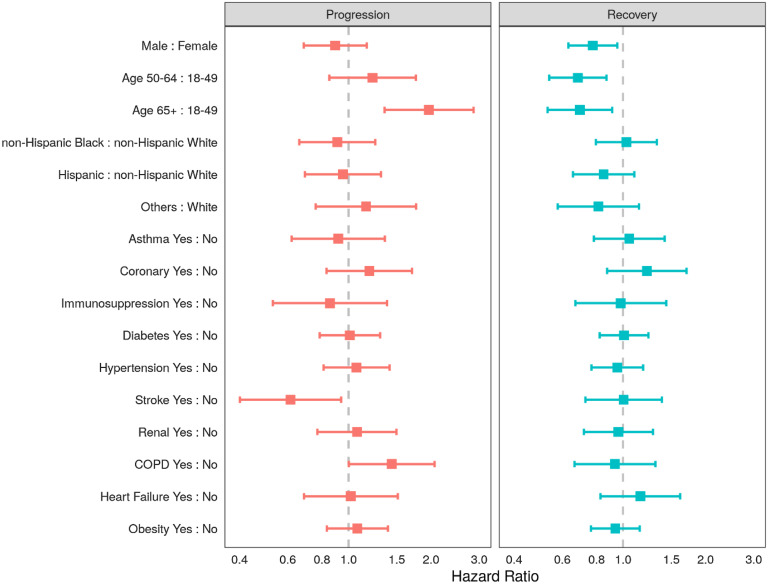
Adjusted hazard ratios of progression and recovery from COVID-19 using a multistate transition model in ptients admitted to the intensive care units (ICUs) in nine participating hospitals, March–July 2020. Note: Adjusting variables included all of the following: sex, age group (18–49, 50–64, 65+ years), race-ethnicity group (non-Hispanic White, non-Hispanic Black, Hispanic, and Other), or the presence of any of 10 comorbidities (asthma, chronic obstructive pulmonary disease, stroke, coronary artery disease, diabetes mellitus, obesity, hypertension, chronic kidney disease, heart failure, or immunosuppression).

Proportional hazards for recovery or progression on any given day are shown in Fig. 2. In the multistate transition model, males and older persons had lower probabilities of recovery transition on any given day: (hazard ratio [HR] for males 0.78, 95% CI 0.63, 0.95); HR 0.68 (95% CI 0.54, 0.87) for the 50–64 years age group and HR 0.70 (95% CI 0.53, 0.91) in the ≥65 age group (reference group: 18- to 49-year-olds). Moreover, those in the oldest age group (65+ years) were more likely to progress (HR 1.97 95% CI 1.35, 2.86) than recover. Among the studied comorbidities, patients with COPD were more likely to progress on any given day compared with patients without COPD (HR 1.44 95% CI 1.00, 2.06). Extensions to this modeling strategy would allow the transition probabilities to vary over time within subgroups and for the introduction of intercurrent events that might even inform state transitions, such as the use of concomitant therapy.

## Discussion

This multisite analysis of data from nine academic ICUs models daily clinical trajectory for critically ill COVID-19 patients early in the pandemic. Our findings are consistent with prior research, showing that male sex [10] and older age [11] are risk factors for poor prognosis in critically ill patients with COVID-19. By showing how the probability of progressing or recovering on any given day depends on baseline covariates, we demonstrate that a multistate transition model based on daily assessments of an ordinal clinical state can offer additional information beyond studies that consider single times or events. This method could prove useful in understanding how therapies affect disease trajectory and may complement traditional clinical trials.

Through this report, we demonstrate how modeling daily assessments could improve our ability to understand the clinical course of critically ill patients with COVID. In the 516 admissions analyzed, we identified over 6,000 daily states reflecting over 5,600 possible state changes. State changes occurred frequently, underscoring the dynamic nature of critical illness in COVID-19. Incorporating temporal information could help to improve our understanding of factors that affect the need for respiratory support in the critical care setting for future pandemics. Further, evaluation of subgroups (e.g., patient demographics or clinical characteristics) is possible and could be pursued to elucidate potential implications for specific patient populations.

This report is intended as a demonstration that the use of transition state modeling provides utility in evaluating the transition of patients between different states of health during critical illness. As such, we have offered a reasonably simplistic treatment of the data. A more thorough treatment of these data would involve exploring the proportionality assumption, accounting for the within-subject and within-site correlation for the repeat admissions, allowing the effects of baseline covariates or treatments to differ depending on the level of the outcome (i.e., level on the clinical scale), to include time-varying covariates (e.g., clinical severity), including differential effects of each covariate, and to explore time by treatment interaction effects. The focus of this work is also restricted to events that occurred in the ICU; states that occurred during the remainder of hospitalizations were not included. A more systematic evaluation of health state after ICU discharge may have allowed us to model the risk of return to the ICU, or return of symptoms and would be recommended. Recognizing that worsening can occur after an improvement is a strength of longitudinal modeling strategies over methods that evaluate time to recovery.

We caution readers not to overinterpret our report; whether the current clinical trajectory of patients in ICUs remains similar is unknown as management practices have changed and additional therapeutic options have become available [12,13]. Such a phenomenon could be explored by considering the period of the pandemic as a covariate in the model We also note that our outcome scale is restricted to a subset of the WHO’s 11-point Clinical Progression Scale [1], which covers the entire spectrum of COVID-19 from undetectable viral levels through death. Given the challenge of obtaining daily observational surveillance data subsequent to ICU discharge, our outcome scale is only comparable to the WHO Clinical Progression Scale for inpatients. Finally, our simplifying assumption allows for the demonstration of this transition model. Removing this assumption could allow for a more thorough understanding of the relationship each covariate has on clinical progression or recovery. Despite the limitations in both the interpretation and scope of our demonstration model, we posit that this approach would be similarly informative if routine assessments were available in any patient setting and for any ordered scale that is reflective of patients’ experience. Indeed, the approach that we describe here is applicable to other diseases, specific subgroups within our current population, to future COVID-19 surges, and to future epidemics and pandemics.

## Conclusions

Using a multistate model of daily assessments, we have demonstrated a method of modeling the dynamic nature of critical illness in COVID-19 patients as it relates to baseline covariates. Future use of methods such as these for modeling change over time could improve our understanding of how therapies and other interventions influence the clinical trajectory of all patients, not just critically ill patients with COVID-19.

## References

[1] W. H. O. Working Group on the Clinical Characterisation Management of Covid-infection. A minimal common outcome measure set for COVID-19 clinical research. The Lancet Infectious Diseases 2020; 20(8): e192–e197. 32539990 10.1016/S1473-3099(20)30483-7 PMC7292605

[2] Douin DJ , Ward MJ , Lindsell CJ , et al. ICU bed utilization during the coronavirus disease 2019 pandemic in a multistate analysis—March to June 2020. Critical Care Explorations 2021; 3(3): e0361. 33786437 10.1097/CCE.0000000000000361 PMC7994039

[3] Peña EA. Dynamic modelling and statistical analysis of event times. Stat Sci 2006 Nov; 21(4): 1–26. DOI 10.1214/088342306000000349. 17906740 PMC1995117

[4] Siebert U , Alagoz O , Bayoumi AM , et al. State-transition modeling: a report of the ISPOR-SMDM Modeling Good Research Practices Task Force-3. Medical Decision Making 2012; 32(5): 690–700. 22990084 10.1177/0272989X12455463

[5] Self WH , Tenforde MW , Stubblefield WB , et al. Seroprevalence of SARS-CoV-2 among frontline health care personnel in a multistate hospital network - 13 academic medical centers, April-June 2020. MMWR. Morbidity and Mortality Weekly Report 2020; 69(35): 1221–1226. 32881855 10.15585/mmwr.mm6935e2 PMC7470460

[6] Lytle KL , Collins SP , Feldstein LR , et al. Influenza vaccine acceptance and hesitancy among adults hospitalized with severe acute respiratory illnesses, United States 2019-2020. Vaccine. 2021; 39(37): 5271–5276. 34376307 10.1016/j.vaccine.2021.07.057 PMC8588478

[7] Jackson C. Multi-state Modelling with R: The msm Package. Cambridge, UK, 2007:, 1–53.

[8] Team RC. R: a language and environment for statistical computing. Published 2021. Accessed. *Available at:* https://www.R-project.org/.

[9] Jackson CH. Multi-State models for panel data: the msm package for R. Journal of Statistical Software. 2011; 38(8): 1–28.

[10] Peckham H , de Gruijter NM , Raine C , et al. Male sex identified by global COVID-19 meta-analysis as a risk factor for death and ITU admission. Nature Communications 2020; 11(1): 6317. 10.1038/s41467-020-19741-6 PMC7726563 33298944

[11] Rosenthal N , Cao Z , Gundrum J , Sianis J , Safo S. Risk factors associated with in-hospital mortality in a US national sample of patients with COVID-19. JAMA Network Open 2020; 3(12): e2029058. 33301018 10.1001/jamanetworkopen.2020.29058 PMC7729428

[12] Beigel JH , Tomashek KM , Dodd LE , et al. Remdesivir for the treatment of Covid-19 - final report. New England Journal of Medicine 2020 Nov 5; 383(19): 1813–1826. DOI 10.1056/NEJMoa2007764. 32445440 PMC7262788

[13] RECOVERY Collaborative Group. Dexamethasone in hospitalized patients with Covid-19. New England Journal of Medicine 2021 Feb 25; 384(8): 693–704. DOI 10.1056/NEJMoa2021436. 32678530 PMC7383595

